# How He Lived so Long

**Published:** 1859-01

**Authors:** Grant Thorburn

**Affiliations:** New Haven


### HOW HE LIVED SO LONG.

Esteemed Friend.

In the article on Tea and Coffee in the Journal for November, you almost describe my manner of life from my youth up. As far back as memory serves, I never could eat, drink or swallow anything with pleasure, hotter or colder than the blood, hence whiskey, rum and brandy, were an abomination in my eyes.

Cold spring water I never drank if I could help it. If memory is correct, about fifty years ago, when looking in a tumbler of water through a microscope, I saw a shoal of small fishes sporting in the water. Thinks I to myself, if we must swallow eels, it may be well to boil them first. From that day cold tea and cold coffee without milk or sugar has been my drink between meals; at breakfast and dinner half a pint of coffee without sugar. I never use milk in either tea or coffee. I eat of all the fruits in their season, but they must first pass through the fire in the shape of pies, tarts or sweet-meats Beets, carrots, and turnips, I eat in moderation; but parsnips are my favorites. Cabbage, lettuce, celery and cucumbers, are proper food for cows. Pickles, and the smell of vinegar, my soul abhorreth. I nursed among the sick seventeen summers, when the yellow fever was in New York; the rooms were always sprinkled with vinegar. Since then, my nose hates sour krout.

Except in hearing, I am not sensible of any decay during the two years just passed. I rise at seven, A. M., breakfast at eight, dine at twelve, M., tea at five, P. M.; never eat between meals; never eat enough. I walk without a staff, sleep without rocking, and eat beef steaks without the help of branby, bitters, or Brandreth's pills. I select the saplings from the wood pile daily; with my buck and saw I cut them in pieces, four inches long. This feeds the stove, warms the room, and drives dull care away.

Thine, with respect.

Grant Thorburn, Senr.,

Aged 85 years and 9 months.

New Haven, Nov. 13, 1858.

